# Counteractions of a Novel Hydroalcoholic Extract from Lens Culinaria against the Dexamethasone-Induced Osteoblast Loss of Native Murine Cells

**DOI:** 10.3390/cells11192936

**Published:** 2022-09-20

**Authors:** Marina Antonacci, Jacopo Raffaele Dibenedetto, Fatima Maqoud, Gerardo Centoducati, Nicola Colonna, Francesco Leonetti, Domenico Tricarico

**Affiliations:** 1 Department of Pharmacy-Pharmaceutical Science, University of Bari “Aldo Moro”, Via Orabona 4, 70125 Bari, Italy; 2 Department of Medicine Veterinary, University of Bari “Aldo Moro”, Str 62 to Casamassima, Valenzano, 70121 Bari, Italy; 3 Terre di Altamura Srl, 70022 Altamura, Italy

**Keywords:** Lens culinaria, chemoprotective, cytotoxicity

## Abstract

The cytoprotective effects of a novel hydroalcoholic extract (0.01–5 mg/mL) from Lens culinaria (Terre di Altamura Srl) were investigated within murine native skeletal muscle fibers, bone marrow cells, and osteoblasts, and in cell lines treated with the apoptotic agent staurosporine (2.14 × 10^−6^ M), the alkylating drug cisplatin (10^−4^ M), the topoisomerase I inhibitor irinotecan (10^−4^ M), the antimitotic pro-oxidant doxorubicin (10^−6^ M), and the immunosuppressant dexamethasone (2 × 10^−6^ M). An amount of 10g of plant material was used to obtain a 70% ethanol/water product, following two-step extraction, evaporation, lyophilization, and storage at −20 °C. For the murine osteoblasts, doxorubicin reduced survival by −65%, dexamethasone by −32% and −60% after 24 and 48 h of incubation time, respectively. The extract was effective in preventing the osteoblast count-reduction induced by dexamethasone; it was also effective at preventing the inhibition of mineralization induced by dexamethasone. Doxorubicin and cisplatin caused a significant reduction in cell growth by −77% for bone marrow cells, −43% for irinotecan, and −60% for dexamethasone, but there was no evidence for the cytoprotective effects of the extract in these cells. Staurosporine and doxorubicin caused a fiber death rate of >−40% after 18 and 24 h of incubation, yet the extract was not effective at preventing these effects. The extract was effective in preventing the staurosporine-induced reduction of HEK293 proliferation and colony formation in the crystal violet DNA staining and the clonogenic assays. It was also effective for the cisplatin-induced reduction in HEK293 cell proliferation. The extract, however, failed to protect the SHSY5Y neurons against cisplatin and irinotecan-induced cytotoxicity. A UV/VIS spectroscopy analysis showed three peaks at the wavelengths of 350, 260, and 190 nm, which correspond to flavonoids, proanthocyanins, salicylates, and AA, constituting the extract. These data suggest the possible development of this extract for use against dexamethasone-induced bone loss and renal chemotherapy-induced damage.

## 1. Introduction

Lens culinaria is a native annual plant whose seed coat contains a large number of flavonoids and proanthocyanidins [1]. It represents an abundant source of protein and provides both essential and non-essential amino acids to the human body. The predominant protein is globulin (47% of total proteins), to which an adequate amount of albumin is added. It is a food relatively low in sodium and fat but with a high quantity of potassium; therefore, it is certainly the most suitable food for patients suffering from obesity and cardiovascular disorders [2]. The evidence shows that the consumption of cooked lentils is associated with a reduction in the incidence of diseases such as diabetes and cardiovascular diseases [3]. Lentil extracts showed peptide-mediated health effects [4] and cholesterol-lowering effects [5]. This legume can stimulate the metabolism of glucose, lipids, and lipoproteins in the blood. A fundamental role is played by the high content of fibers and flavonoids that stimulate intestinal motility, meaning the regular consumption of cooked lentils in diabetic patients leads to a significant reduction in fasting blood sugar, glycemic load, and glycemic index. Lentils are also a source of iron, and it is known that the consumption of this cooked legume prevents iron deficiency anemia. They contain many minerals (zinc, iron, manganese, selenium, and boron) and vitamins (thiamin, riboflavin, niacin, pantothenic acid, pyridoxine, folate and tocopherols, and phylloquinone). On the other hand, they have a low content of vitamin K, making them a safe food for patients with cardiovascular diseases in treatment with anticoagulants [3]. Moreover, the polyphenol content has the potential to reduce blood pressure through an inhibitory activity on the enzyme responsible for converting angiotensin I (ACE). Recent studies have observed how the bioactive compounds (legumin, vicillin, and convicillin) of lentils possess antioxidant, ACE-inhibitory, and cardioprotective activity, resulting in a reduction in the risk of hypertension and coronary heart disease [6]. On the other hand, studies on the possible preventive activity of lentils on osteoporosis are poor. The in vivo treatment of ovariectomized rats that develop a high osteoporotic risk with a lentil-based soup improved their bone morphology by preventing osteoporosis [7]. Finally, the consumption of lentils appears to reduce the incidence of various types of cancer, including colon, thyroid, breast, and prostate cancer. A large prospective epidemiological study that associated polyphenol-rich lentils with breast cancer showed an inverse correlation between lentil consumption and the risk of developing the disease [8]. However, whether these anti-cancers effects are directly mediated by cytoprotective actions or mediated by indirect effects on survival is not known. It has been proposed that the high polyphenolic content of these legumes could prevent carcinogenesis through chemo-preventive activity, preventing the oxidative damage of DNA induced by chemotherapy [9], suggesting direct cytoprotective action.

So, considering the known positive role of legumes on human health and sustainable agriculture systems, and considering that 2016 has been declared the “International Year of legumes” by FAO (UN A/RES/68/231), to promote their nutritional use, we investigated the cytoprotective effects of a new hydroalcoholic extract of culinary Lens of Apulian origin (02.170221.96 Terre di Altamura Srl/TDA) in in vitro cell models.

We evaluated the biological activity of a novel extract (1E030521DIPFARMTDA) in the prevention of the cytotoxic damage caused by chemical agents that act with different mechanisms, such as staurosporine, a non-selective tyrosine kinase inhibitor, and irinotecan, cisplatin, and doxorubicin. Staurosporine is an alkaloid isolated from Streptomyces staurospores culture broth, mainly used in the past as a protein kinase C inhibitor, but which targets several other tyrosine kinases (TK), so it is considered a cell-permeable and broad-spectrum TKI. The main biological activity of staurosporine is associated with the inhibition of protein kinases through the prevention of the interaction of ATP at the kinase site. Staurosporine is a prototype ATP-competitive inhibitor as it binds to many kinases with a high affinity but lacks selectivity. Indeed, it has been shown that staurosporine inhibits cell cycle progression in a large variety of cell lines and inhibits the invasion of tumor cells [10]. Besides this effect on the cell cycle, it is also an ion channel modulator [11]. Chemotherapeutics, like irinotecan and SN-38, its active metabolite and a molecule that inhibits topoisomerase I, which is the enzyme responsible for the breaking-re-stitching of a single DNA strand, were also investigated in this work. The inhibition of this enzyme leads to genomic aberrations and the consequent triggering of apoptosis and cell death. Irinotecan is an anticancer substance commonly used for the first-line treatment of pancreatic cancer and in the second-line treatment of colorectal cancer. However, it causes a series of secondary reactions, such as diarrhea and, above all, neutropenia [12]. Cisplatin, instead, is an antineoplastic chemotherapy alkylating agent binding to DNA through the formation of cross-links between complementary strands, able to interfere with all phases of the cell cycle. Finally, doxorubicin is an antimitotic that binds cellular DNA, which inhibits nucleic acid synthesis and mitosis, causing chromosomal aberrations. This drug has made it possible to improve the prognosis of children suffering from neuroblastoma, sarcoma, hepatobiliary, head, neck, and testicular cancer. However, it causes significant toxicity, which includes ototoxicity, nephrotoxicity, neurotoxicity, and myelotoxicity. The incidence of these adverse reactions is related to factors such as the patient’s age and the presence of co-morbidities, and is also based on the administered dose [13]. Doxorubicin is not a phase-specific molecule but is active above all in the S phase of the cell cycle, inducing oxidative stress and cytotoxic effects in various types of cells, including osteoblasts. Even the bone marrow, the oral and gastrointestinal mucosa, and the hair bulbs (all rapidly proliferating cells) are affected by doxorubicin activity. All these drugs are therefore used in various chemotherapy protocols during the treatment of solid and liquid tumors and cause bone marrow toxicity with depression associated with neutropenia, anemia, and lymphocytopenia, bone cytotoxicity with metastases, and antiproliferative effects on various types of renal and intestinal cells in active proliferation. Neuromuscular toxicity has also been observed in platinum derivatives and other chemotherapeutic agents [14]. These adverse reactions can be dose-limiting, preventing the achievement of the therapeutic dose and forcing the clinician to switch to another protocol in many cases with therapeutic failure. A relevant research area is the development of chemo-preventive agents to be co-administered with chemotherapeutic agents to prevent adverse drug reactions.

In addition, we evaluated the in vitro capability of the extract in counteracting osteoblast loss following corticosteroids like dexamethasone. Osteoporosis is one of the serious complications of oral corticosteroid treatment. Several studies report a decrease in bone mineral density irrespective of the disease being treated [15,16,17,18]. A meta-analysis shows that more than 5 mg/daily treatment with oral corticosteroids leads to a reduction in bone mineral density and a rapid increase in the risk of fracture, and this is observed also with inhaled corticosteroids [19,20,21]. Adverse drug reactions (ADR) from the musculoskeletal apparatus are reported with corticosteroids [22].

In this work, the chemo-preventive effects of a novel hydroalcoholic extract were investigated in vitro on a primary cell model culture used in our labs and derived from mouse bone marrow, osteoblast cells, and isolated skeletal muscle fibers to evaluate the efficacy of this extract to prevent osteoblast loss, bone marrow toxicity, and the musculoskeletal atrophic effects induced by chemotherapy drugs and dexamethasone. Experiments were also performed on HEK293 renal and SHSY5Y neuronal cell lines. The effect of diazoxide, a modulator of ATP-dependent potassium channels (KATP) and an activator of mitochondrial oxidative phosphorylation already used in the clinic as a hyperglycemic, neuroprotector, and anti-atrophic drug [11,23], was also investigated. The biological activity was related to the phyto-complex composition and to the ability of these substances to interact with known molecular targets, such as estrogen receptors [24], and new molecular targets relevant in osteoblast genesis processes through in silico and in vitro studies [25,26].

## 2. Materials and Methods

### 2.1. Preparation of a Novel Hydroalcoholic Extract

Lens culinaria powder was obtained by finely grinding lentil seeds supplied by Terre di Altamura Srl (BA) in a metal blade blender to obtain a homogeneous powder of minimum particle size. For the hydroalcoholic extract, 10 g of plant material was weighed, placed in a 50 mL plastic tube, and extracted with 50 mL of 70% ethanol/water for 1 h in a shaker at room temperature. The residue was re-extracted one more time under the same condition overnight (16 h). The next day, both extracts were filtered through paper filters (Whatman Filter papers diameter 110 mm), evaporated at 35 °C under reduced pressure in Rotavapor, then further lyophilized, resuspended in PBS, and stored at −20 °C. The concentration range tested was 0.01 mg/mL to 5 mg/mL at T = 37 °C for 3–96 h incubation periods. The preparation of the extract and the product under investigation is new and not previously described.

#### UV/VIS Spectroscopic Analysis

The variable wavelength spectroscopic analysis in scan mode was carried out using a Varian 50 spectrophotometer (Varian Cary Inc., Agilent Technologies Italy S.p.A., Milan, Italy) on samples with increasing dilutions in water. The UV/VIS spectra were used to monitor the stability of the extract before testing in the cell culture.

### 2.2. Drugs Solution

The drugs were purchased from Sigma (SIGMA Chemical Co., Milan, Italy). Stock solutions of the drugs under investigation were prepared by dissolving the compounds in dimethylsulfoxide (DMSO); the stock solution of staurosporine was prepared at a concentration of 5 mg/mL which was diluted in DMEM at a 21.4 × 10^−6^M concentration, to give a final concentration of 2.14 × 10^−6^ M (staurosporine) in the wells; the stock solution concentration of diazoxide was 118.6 × 10^−3^ M which diluted in DMEM at 250 × 10^−5^ concentration to give a final concentration of 250 × 10^−6^ M (diazoxide) in the wells; the stock solutions concentrations of cisplatin and irinotecan was 0.5 M which was diluted in DMEM at 1 × 10^−3^ M concentration to give a final concentration of 1 × 10^−4^ M (cisplatin and irinotecan); the stock solution concentration of doxorubicin was 86.2 × 10^−3^ M which was diluted in DMEM at 100 × 10^−3^M concentration to give a final concentration of 1 × 10^−6^ M (doxorubicin) in the wells; for the dexamethasone the stock solution concentration was 0.25 M which was diluted in DMEM at 2 × 10^−5^ M concentration to give a final concentrations of 2 × 10^−6^ M (dexamethasone) in the wells. Microliter amounts of the stock solutions were then added to DMEM+, for crystal violet staining, MTT, clonogenic assay, and direct fiber count by visual inspection [11,27]. DMSO concentration does not normally affect the parameters under study. The extract was tested under a wide range of concentrations (0.01–5 mg/mL) commonly used in these experiments; concentrations higher than 5 mg/mL lead to an unstable solution in the culture medium monitoring UV/VIS spectra in the culture medium.

### 2.3. Cell Culture

The tissues for the preparation of native cells were explanted from mice (WT/WT) and used for other experimental purposes [28]. Animal care and all experimental protocols are in agreement with the European Directive 2010/63/EU on Animal Protection Used for Scientific Experiments and were approved by the Italian Ministry of Health and by the Committee of the University of Bari, (prot. 8515-X/10, 30-January-2019). Bone marrow cells and osteoblasts were obtained from mouse tibia and femur tissue [26]. The bones were dislocated, separated, washed with stepwise dilutions of ethanol, and finally placed in a Petri dish in PBS. Subsequently, we collected the bone marrow, perfusing a sterile solution of PBS inside the medullary cavity, using a 5 mL syringe [29]. The entire procedure was conducted under sterile conditions to prevent contamination. The cells obtained were cultured in Dulbecco’s Modified Eagle’s Medium (DMEM) enriched with 10% fetal bovine serum (FBS), 1% L-glutamine, and 1% antibiotics (penicillin\streptomycin) and placed in a standard incubator. Non-adherent cells were carefully removed after 3 h of incubation.

The enrichment of the culture with mesenchymal stem cells (MSC) was performed as follows: after primary cultures had become almost confluent, they were treated using Trypsin-EDTA Solution 1× (0.25% trypsin, 0.02% EDTA) for 2 min at room temperature. The same protocol was applied 3 times so that a purified population of MSCs was obtained 3 weeks after the initiation of culture, and then used for mineralization experiments. Mouse bone-marrow-derived osteoblasts for the mineralization assay were then cultured in the mineralization medium obtained by the enrichment of DMEM with 50 µg/mL ascorbic acid and 10 mM β-glycerophosphate (Sigma-Aldrich, St. Louis, MO, USA). Primary bone cells can produce extracellular matrix, as revealed by Alizarin red staining, confirming the functional osteoblastic phenotype. The cell pellets were positive for Alkaline phosphatase (*ALP*), Osteocalcin (*BGLAP*), and Msh homeobox 2 (*MSX2*) genes in PCR analysis, as previously shown [30].

Muscle fibers were prepared from Flexor digitorum brevis (FDB) muscles by enzymatic dissociation in normal Ringer solution, as previously described [31,32,33]. After enzymatic dissociation, the isolated fibers were used for morphology evaluation.

The 293 human embryo kidney-derived [HEK-293] cell line, with epithelial morphology (ATCC), and SHSY5Y CRL-2266 (ATCC) cell line of the neuroblastoma. These cells were cultured in Minimum Essential Medium Eagle-Alpha Modification (αMEM) supplemented with 10% fetal bovine serum (FBS), 1% L-glutamine, and 1% antibiotics (penicillin-streptomycin), at 37 °C in a humidified atmosphere containing 5% CO_2_. The experiments were performed on undifferentiated cells in a passage comprised between P6 and P14 during the experiments.

### 2.4. Mineralization Assay

This assay has been performed to evaluate how osteoblast capability to mineralize is changed by the presence of dexamethasone (2 × 10^−6^ M) alone or in the presence of dexamethasone and the extract. Murine bone marrow cells (1 × 10^5^ cells/mL/well) were cultured for 2 days in 12- or 24-well plates in mineralization medium in the presence or absence of drugs at different concentrations for 10–15 days under standard conditions; the medium was changed every 3 days by adding fresh medium. After culturing, cells were stained with Alizarin red following the same protocol we previously described [26]. The experiment was carried out in triplicate.

### 2.5. Crystal Violet Staining

Cells, previously counted using the Scepter 2.0 counter (Merck KGaA, Darmstadt, Germany), were plated in a multi-well plate with a density of 8 × 10^3^ cells/well. After 24 h of incubation, different concentrations of extract and chemotherapeutic agents were added. After the established incubation time (3, 6, 24, 48, or 72 h) the medium was removed, and the cells were fixed with 10% buffered formalin for 20 min at room temperature and subsequently stained using a 1% solution *v/v* of crystal violet for 30 min. Plates were washed with distilled water to remove excess dye. Finally, acetic acid was added to elute the dye, and the absorbance at 560 nm was read using the Victor^TM^ spectrophotometer (Perkin Elmer, Waltham, MA, USA). Each experimental condition was evaluated in triplicate at least.

### 2.6. Clonogenic Assay

Cells (Number of cells = 250), previously counted using the Scepter 2.0 counter, were plated before treatment in 60 mm plates. The cells were initially kept in the incubator for 24 h. Then, we evaluated the effect of the substances on the number of forming colonies after 3 h of incubation. Each experimental condition was performed in triplicate. After the incubation, the culture medium with the cytotoxic compounds and the extract was removed and replaced with a fresh medium. Cells were then kept in culture for 2 weeks. At the end of the test, the colonies formed were fixed with 10% buffered formalin for 1 h at room temperature and stained with 0.05% *v/v* of crystal violet. To count the number of colonies formed after the incubation time, the use of the OpenCFU 3.9.0. (My Biosoftware, USA) was used, which was accompanied by the manual count of three operators, from which an average was then calculated [34].

### 2.7. MTT Assay

This assay is based on the use of a yellow-coloured salt, the bromide of 3-(4,5 dimethylthiazol-2-yl)-2,5-diphenyltetrazolium (SIGMA-ALDRICH, Mi). An adequately weighed quantity was solubilized in PBS (5 mg/mL), stored at −20 °C and protected from light sources. After plating cells in multi-well plates, followed by appropriate treatments, we proceeded to aspirate the culture medium containing the substances to be tested, and the subsequent addition of 190 μL of DMEM + 10 μ of MTT per well, was to be carried out strictly in the dark. The plate was then incubated at 37 °C and 5% CO_2_ for about 3 h. In the end, the cells were lysed with a 1:1 mixture of dimethyl sulfoxide (DMSO) and 96% (*v***/***v*) ethanol by volume equal to 150 μL per well. The subsequent spectrophotometric reading of the plate was carried out at the Victor^TM^ at a wavelength of 570 nm.

### 2.8. Fiber Survival Evaluation

The effects (0–24 h) of the hydroalcoholic extract against staurosporine and doxorubicin were tested, also on the morphology of the fibers. Evaluation of the morphological parameters of FDB fibers was performed by seeding fibers in the culture medium (DMEM supplemented with 10% fetal bovine serum, 1% L-glutamine, and 1% penicillin-streptomycin), at 37 °C. Isolated fibers were equilibrated in the culture medium for at least 30 min at 37 °C. Dead fibers were defined as cells showing marked changes of ≥40% in morphological parameters such as length and diameter within 24 h from the excision under microscopic evaluation. The appearance of multiple sarcolemma blebs and vacuoles preceded cellular death [28,35,36,37]. On isolated native fibers, death was expressed as the degree of mortality. The first degree of mortality indicates a mortality rate of 0–25%, the second degree indicates a mortality rate of 25–50%, the third degree of mortality indicates a mortality rate of 50–75%, and the fourth degree of mortality indicates a mortality rate of 75–100% [36,38].

### 2.9. Data Analysis and Statistics

The data obtained were collected and analyzed using Excel software (Microsoft Office 2010, Mi, Italy). The results were presented as the mean ± standard deviation. The significance of the results was assessed by performing a one-factor variance analysis using Excel software (Microsoft Office 2010) and statistical significance was attributed to *p* values < 0.05. For the clonogenic test, the plating efficiency (PE) and the survival fraction (SF) were calculated using the clonogenic survival test with the following equations:

(1) $$PE\;\left(\%\right)=\frac{N\;\mathrm{formed}\;\mathrm{colonies}}{N\;\mathrm{plated}\;\mathrm{cells}}\times 100$$

(2) $$\mathrm{SF}=\frac{N\;\mathrm{formed}\;\mathrm{colonies}\;\mathrm{after}\;\mathrm{treatment}\;}{N\;\mathrm{plated}\;\mathrm{cells}\;x\;\mathrm{PE}}\;$$

## 3. Results and Discussion

### 3.1. Evaluation of the Protective Efficacy of the Extract against the Cytotoxic Agents on Murine Primary Bone Marrow Cells, Osteoblast, and Skeletal Muscle Fibers

Considering the bone marrow toxicity caused by chemotherapies, the extract was tested on primary cell cultures obtained from mice femur and tibia. Doxorubicin is an effective chemotherapeutic agent used for the treatment of a variety of cancers, such as breast and stomach cancers, and in the treatment of leukemia. Reports have been made of several side effects, such as nausea, vomiting, and adverse reactions, such as cardiotoxicity and myelosuppression, limiting its use. Doxorubicin is known to interfere with DNA replication by inhibiting topoisomerase II and inducing oxidative stress [37]. So, we investigated for a possible protective effect of the extract against doxorubicin toxicity in bone marrow cells.

**Figure 1:**
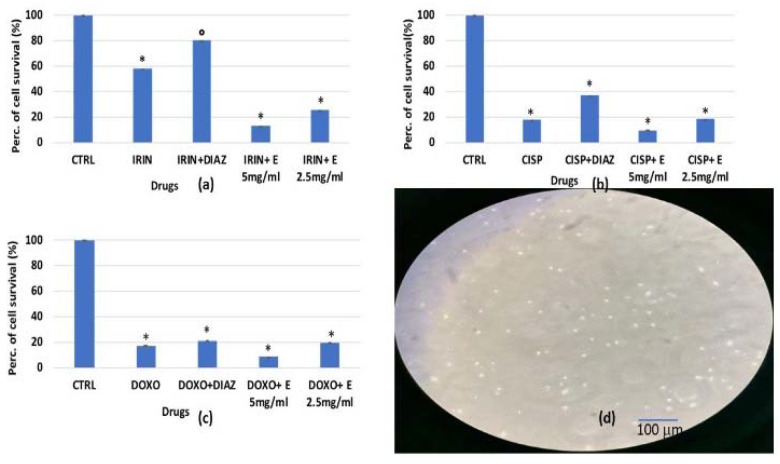
Percentage of the cell growth values of murine bone marrow cells after 96 h of incubation with (**a**) irinotecan 1 × 10^−4^ M (IRIN), (**b**) cisplatin 1 × 10^−4^ M (CISP), (**c**) doxorubicin 2 × 10^−6^ M (DOXO) and diazoxide 2.5 × 10^−4^ M (DIAZ) or different concentrations of the extract (E) measured via crystal violet assay. (**d**) Image of murine bone marrow cell culture at 50% confluency. The reported values represent an average of at least four replicates. One-way analysis of variance with ANOVA was performed to determine the significance of the data vs. controls (CTRL) * or vs. cytotoxic compounds ° with a value of *p* < 0.05.

In this cell culture (Figure 1d), doxorubicin and cisplatin caused a significant reduction in cell growth of −77% in bone marrow cells, while irinotecan reduced the cell growth by −43%; unfortunately, there was no evidence for the cytoprotective effects of the extract against the three chemotherapeutic drugs tested after 96 h of incubation (Figure 1a–c). Only diazoxide showed a protective effect against irinotecan-induced bone marrow cytotoxicity (Figure 1a).

We also investigated the effects of the extract over a shorter incubation time of 48 h against doxorubicin, cisplatin, and irinotecan. Despite a reduction in incubation times, the extract was still not effective in counteracting bone marrow cell depression induced by the chemotherapeutic drugs even after 48 h of co-incubation with these drugs. In addition, dexamethasone, a well-known immunosuppressant, caused a marked reduction in cell survival of −60% but in this case, the extract was not effective in counteracting the dexamethasone-inducing bone marrow cell depression.

Therefore, the extract did not prevent the cytotoxic damage induced by cisplatin, doxorubicin, dexamethasone, and irinotecan on the primary murine bone marrow cultures.

The effectiveness of the extract was therefore evaluated on osteoblasts responsible for maintaining bone volume and density during the remodeling phases of this tissue in bone metastases and osteoporosis. Additionally, in this case, we were investigating the efficacy of the extract in preventing cytotoxic damage induced by doxorubicin, cisplatin, and dexamethasone.

**Figure 2:**
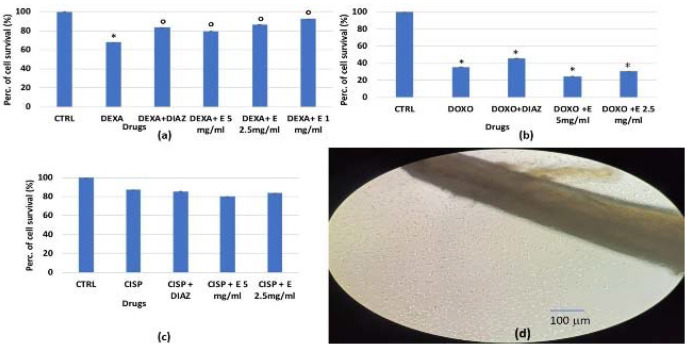
Percentage change in cell growth values for osteoblasts after 24 h of incubation with: (**a**) dexamethasone 2 × 10^−6^ M (DEXA), (**b**) doxorubicin 1 × 10^−6^ M (DOXO), (**c**) cisplatin 1 × 10^−4^ M (CISP), and diazoxide (DIAZ) (250 × 10^−6^ M) or the extract (E) at different concentrations in the crystal violet assay. (**d**) Image of murine osteoblasts migrating from bone fragments at 90% confluency. The reported values represent an average of at least six replicates. One-way analysis of variance with ANOVA was performed to determine the significance of the data vs. controls (CTRL) * or cytotoxic compounds ° with a value of *p* < 0.05.

All of these drugs caused a reduction in cell proliferation, especially doxorubicin, which reduced survival by −65% compared to the controls, and dexamethasone, which reduced it by −32%. Only a mild cytotoxic effect was observed for cisplatin. The extract was found to be significantly effective in preventing cell reduction induced by dexamethasone but not by doxorubicin (Figure 2a–c).

**Figure 3:**
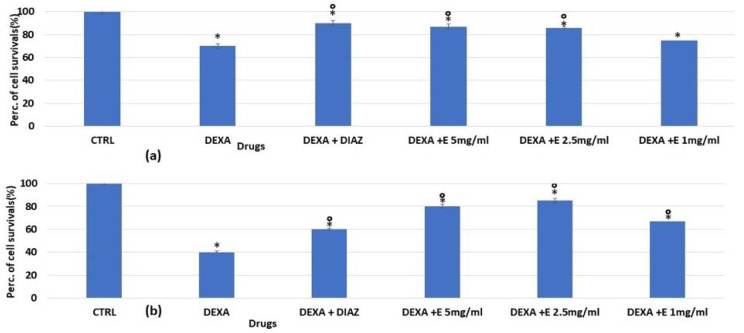
Percentage changes in cell growth values for osteoblasts after (**a**) 24 h and (**b**) 48 h of incubation with dexamethasone 2 × 10^−6^ M (DEXA), dexamethasone, and diazoxide (DIAZ) (250 × 10^−6^ M), and dexamethasone in the presence of a different concentration of the extract in the MTT assay. The reported values represent an average of at least six replicates. One-way analysis of variance with ANOVA was performed to determine the significance of the data vs. controls (CTRL) * or dexamethasone ° with a value of *p* < 0.05.

We then evaluated the effect of the extract against dexamethasone after 24 and 48 h of incubation time in murine osteoblasts via MTT assay (Figure 3a,b). We found that the extract was fully effective in preventing the dexamethasone-induced reduction of osteoblast counts (also using this method), suggesting the possible involvement of mitochondrial dehydrogenase activity in its cytoprotective actions.

**Figure 4:**
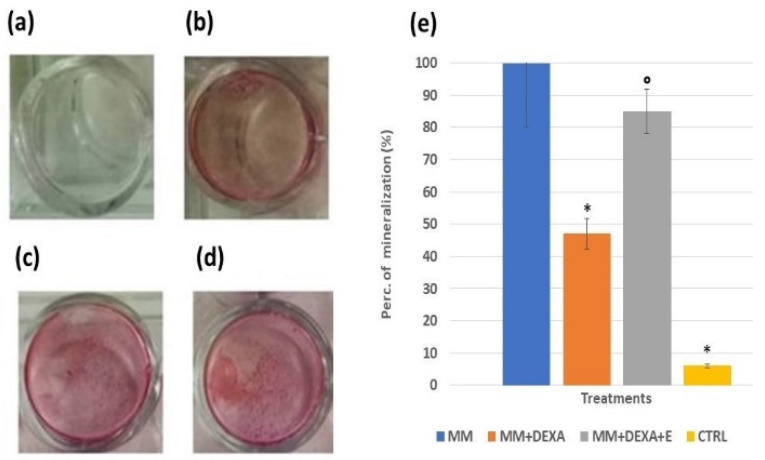
Mineralization assay with Alizarin red staining for calcium nodules upon treatment with the extract (2.5 mg/mL) in the presence or absence of dexamethasone (DEXA) (2 × 10^−6^ M) after 15 days of incubation in osteoblasts-derived murine bone marrow cells, following differentiation. (**a**) Control condition of osteoblasts in normal medium, (**b**) DEXA in the presence of the mineralization medium (MM), (**c**) DEXA and the extract in the presence of MM, (**d**) osteoblasts culture in the presence of MM, (**e**) histogram showing the cytoprotective effect of the Extract against the DEXA-induced reduction of the MM, the data were from at least three replicates. One-way analysis of variance with ANOVA was performed to determine the significance of the data vs. controls (CTRL) * or dexamethasone ° with a value of *p* < 0.05. Images were captured using an Olympus CX41 biological system microscope at 40× magnification.

In another series of experiments, we evaluated the capability of the extract at a 2.5 mg/mL concentration to counteract the reduction of mineralization induced by dexamethasone. Dexamethasone (2 × 10^−6^ M) indeed reduced mineralization by −53% (Figure 4b,d) for the bone marrow differentiated osteoblasts in the presence of mineralization medium (MM) in our experiments vs. the MM conditions. However, we found that the extract was fully effective in counteracting this effect (Figure 4c,d)

**Figure 5:**
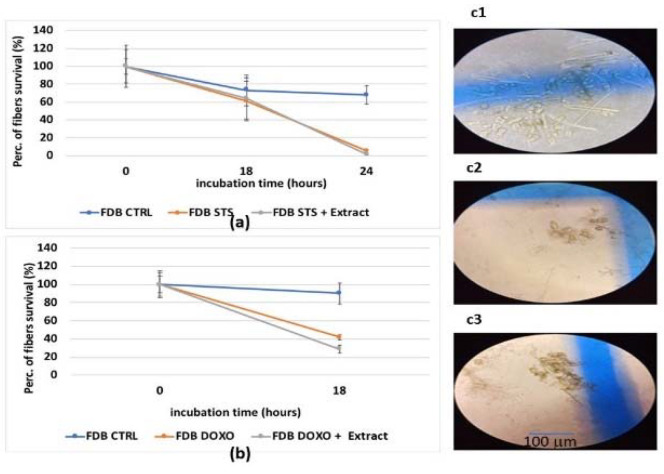
(**a**) Evaluation of FDB fiber survival after an incubation time of 18 and 24 h with staurosporine (STS) (2.14 × 10^−6^ M) and the extract (2.5 mg/mL) (E). (**b**) Evaluation of FDB fiber survival after an incubation time of 18 h with doxorubicin (1 × 10^−6^ M) and extract. Image of FDB fibers (**c1**) in the control condition (CTRL) after 24 h of incubation time, where cell death and alive fibers are observed and counted (**c2**) after 18 h incubation time with doxorubicin (**c3**) after 24 h of incubation time with staurosporine. A blind evaluation of the morphological parameters of the fibers was performed. The fiber survival was evaluated by a direct visual inspection and cell counting under a Zeiss Axiovert 10 inverted microscope (×10). For each fiber, three individual measurements were performed at three or two different points. The appearance of multiple sarcolemma blebs often preceded cellular death.

For FDB isolated murine muscle fibers, the hydroalcoholic extract was also not effective in preventing the cell death caused by staurosporine and doxorubicin (Figure 5a,b). Despite co-incubation with the extract, staurosporine caused a death rate of −40% after 18 h of incubation, reaching 100% after 24 h of incubation (Figure 5a), as previously reported [11,28,38]. Doxorubicin caused a death rate of −60% after 18 h of incubation and −80% death rate after 24 h of incubation (Figure 5b). Additionally, in this case, the extract was not effective in preventing fiber death (Figure 5b,c1–c3).

### 3.2. Evaluation of the Extract’s Efficacy in Preventing the Staurosporine (STS)-Induced Cytotoxicity in Cell Lines

No effects of the extract were observed at all concentrations tested on cell growth on the renal HEK293 and neuronal SHSY5Y cell lines in the crystal violet assay (Figure S1).

We, therefore, evaluated the possible cytoprotective effect of the extract on cells co-incubated with a cytotoxic substance, such as staurosporine at a concentration of 2.4 × 10^−^^6^ M. The cells were plated at a density of 8 × 10^3^ cells/well and coincubated with the same volume of staurosporine and extract solutions that were added to the same wells. Diazoxide, a well-known KATP channel opener, showed cytoprotective and anti-atrophic action against staurosporine-induced cytotoxicity in isolated fibers and cell culture in previous works [11,38]. 

**Figure 6:**
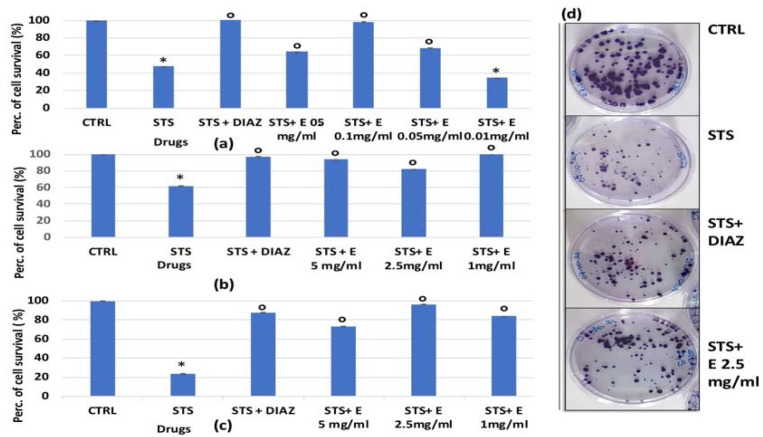
Percentage of cell growth values for HEK293 cells co-incubated with staurosporine 2.14 × 10^−6^ M (STS) and diazoxide 250 × 10^−6^ M (DIAZ) or various concentrations of the extract (E) measured with crystal violet assay vs. controls (CTRL). Panels: (**a**) show data at lowest concentrations of the extract (0.5 mg/mL, 0.1 mg/mL, 0.05 mg/mL and 0.01 mg/mL) after 6 h of incubation. Panels: (**b**,**c**) show data at highest extract concentrations (5 mg/mL, 2.5 mg/mL, and 1 mg/mL) after 3 and 6 h of incubation, respectively. The reported values represent an average of at least seven replicates. (**d**) The effect of STS 2.14 × 10^−6^ M, diazoxide 250 × 10^−6^ M, and extract (2.5 mg/mL) on cell clone formation was evaluated after 3 h of incubation measured in the clonogenic assay. One-way analysis of variance with ANOVA was performed to determine the significance of the data vs. controls * or cytototoxic ° compounds with a value of *p* < 0.05.

On human renal HEK293 cells, staurosporine caused a significant reduction in cell proliferation, with percentages ranging from −40% to −80% (*p* < 0.05), and the extract was significantly effective in counteracting this reduction (*p* < 0.05), with activity comparable to that of diazoxide (Figure 6a–c). After 6 h of incubation, the effects of the extract were concentration-dependent (Figure 6a), while under a shorter incubation time, the extract at low concentrations partly prevented the reduction of cell number induced by STS.

In the clonogenic assay, we evaluated the possible protective effect of the novel Lens c. hydroalcoholic extract at a 2.5 mg/mL concentration on the ability of the cells to form colonies after 3 h of incubation with staurosporine (2.14 × 10^−6^ M). The protective activity of the extract was compared with that of diazoxide (250 × 10^−6^ M).

We found that, for the HEK293 renal cell line, staurosporine significantly reduced the number of cell colonies after 3 h of incubation time. The extract was capable of counteracting this effect. The cytoprotective efficacy of the extract towards staurosporine was comparable with that of diazoxide (also in the clonogenic assay). The surviving fraction of the colonies was indeed 0.585 ± 0.01 after STS alone (*p* < 0.05), 0.7138 ± 0.03 (Number of test = 3, *p* < 0.05), and 0.831 ± 0.04 after STS+diazoxide (Number of test = 3, *p* < 0.05) and STS + the extract (Number of test = 3), respectively (Figure 6d).

On the other hand, for the SHSY5Y neuronal line, staurosporine is not effective in reducing cell proliferation, always maintaining values close to 100% for cell proliferation, with this being in line with the recognized role of this compound in the neuronal differentiation of this cell line; by itself, it has no cytotoxic effects on these cells [39] (Figure S2).

### 3.3. Evaluation of the Efficacy of the Extract in Preventing the Cytotoxicity Induced by Chemotherapeutic Drugs in Cell Lines

**Figure 7:**
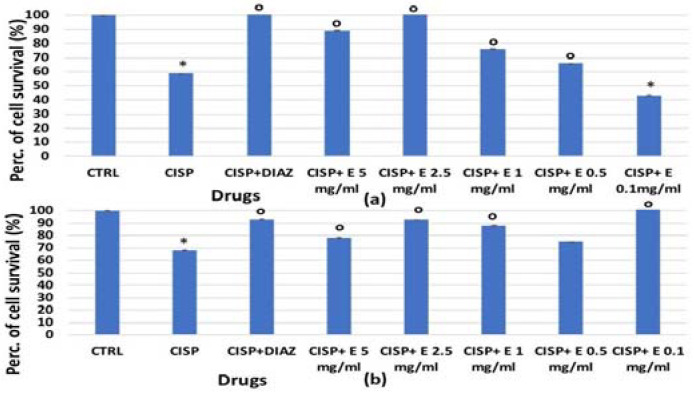
Percentage of cell growth values for the HEK293 cell line co-incubated with cisplatin 1 × 10^−4^ M (CISP) after (**a**) 48 and (**b**) 72 h of incubation time, and diazoxide 250 × 10^−6^ M (DIAZ) or various extract (E) concentrations in the crystal violet assay. The reported values represent an average of at least six replicates. One-way analysis of variance with ANOVA was performed to determine the significance of the data vs. controls * (CTRL) or cytotoxic ° compounds with a value of *p* < 0.05.

The extract’s efficacy was also evaluated against cisplatin and the irinotecan-induced reduction of cell proliferation. For HEK293, a significant reduction in the cell number was observed after 48 and 72 h of incubation time with cisplatin, which caused a significant reduction in cell proliferation of −40% (Figure 3a) and −35%, respectively (Figure 7b). The diazoxide was capable of restoring these values to the control levels. Additionally, the extract has shown effectiveness against the cisplatin-induced reduction of cell proliferation; the effects of the extract at a 2.5 mg/mL concentration were comparable to that of diazoxide. The effect of the extract was concentration-dependent after 48 h of incubation time (Figure 7a).

No significant change in the cell growth values was observed in the presence of the irinotecan on renal HEK293 cells after 48 and 72 h of incubation time, as well as in the presence of increasing concentrations of the extract or with diazoxide, which did not cause a change in the cell proliferation.

**Figure 8:**
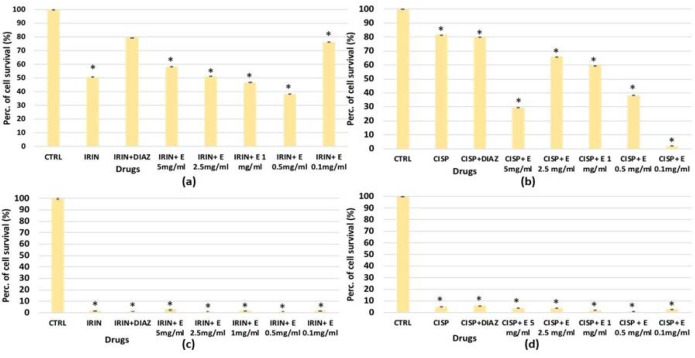
Percentage of cell growth values for the SHSY5Y cell line co-incubated with (**a**) irinotecan 1 × 10^−4^ M (IRIN), (**b**) cisplatin (CISP) 1 × 10^−4^ M and diazoxide 250 × 10^−6^ M (DIAZ) or various extract (E) concentrations after 48 h of incubation time, measured in the crystal violet assay. Panel (**c**) SHSY5Y cell line co-incubated with irinotecan 1 × 10^−4^ M, or (**d**) cisplatin 1 × 10^−4^ M, in the presence of diazoxide 250 × 10^−6^ M (DIAZ) or various extract (E) concentrations after 72h of incubation time. The reported values represent an average of at least six replicates. One-way analysis of variance with ANOVA was performed to determine the significance of the data vs. controls (CTRL) with a value of *p* < 0.05 *.

For SHSY5Y, significant cytotoxicity was observed after 48 h of incubation with cisplatin and irinotecan, which resulted in a significant reduction in cell proliferation, respectively, of –20% and −50% (Figure 8a,b). The diazoxide was not effective against the cisplatin-induced reduction in cell proliferation; on the contrary, it was effective in preventing the cell count reduction induced by irinotecan (Figure 8a). After 72 h of incubation time, either irinotecan or cisplatin caused a −100% reduction in cell proliferation, and neither diazoxide nor the extract showed a cytoprotective effect against both chemotherapeutic agents (Figure 8c,d).

Therefore, the extract prevented the reduction in cell proliferation induced by cisplatin and staurosporine for the renal HEK293 cells in the crystal violet assay. The extract, however, failed to protect the cells against cisplatin- and irinotecan-induced cytotoxicity for the SHSY5Y neuronal cells at all incubation times. Diazoxide was effective as a cytoprotective drug on renal HEK293 cells and against the irinotecan-induced neuronal cell death only after 48 h of incubation time but was largely ineffective against cisplatin-induced neurotoxicity in SHSY5Y neuronal cells.

The observed cytoprotective effects of the extract in HEK293 cells against chemotherapeutic drugs were also evaluated in the MTT assay, in which tetrazolium salt undergoes a reduction in insoluble purple formazan by the enzyme dehydrogenase, located at the mitochondrial level of the viable cells [40]. Therefore, while the crystal violet assay highlighted the DNA of the viable cells and their membrane integrity, the clonogenic assay measures the formation of new clones, and the MTT test assesses the reduction of the cell viability induced by mitochondrial-based dysfunction.

**Figure 9:**
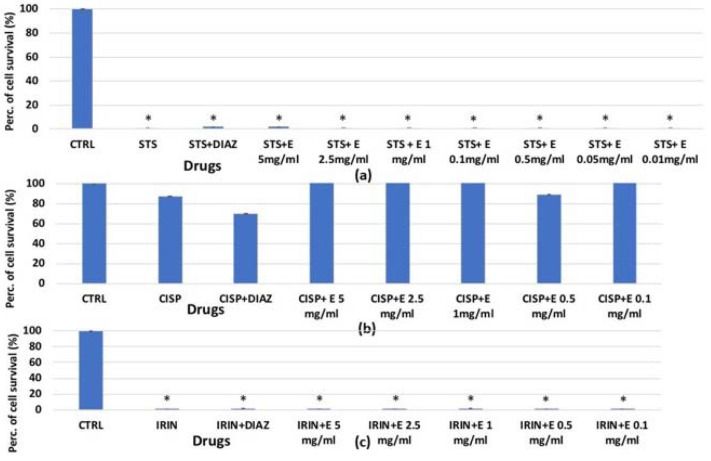
Percentage of cell growth values for HEK293 cells co-incubated (**a**) 6 h with staurosporine 2.14 × 10^−6^ M (STS); (**b**) 72 h with cisplatin 1 × 10^−4^ M (CISP); (**c**) 72 h with irinotecan 1 × 10^−4^ M (IRIN) and diazoxide 250 × 10^−6^ M (DIAZ) or different extract concentrations (E) measured via MTT assay. The reported values represent a mean of at least four replicates. One-way analysis of variance with ANOVA was performed to determine the significance of the data vs. controls (CTRL) with a value of *p* < 0.05 *.

In the MTT assay (Figure 9a), after 6 h of incubation time, staurosporine resulted in a reduction in cell survival of −100% but the extract, and also diazoxide, were not effective in preventing this reduction in contrast to what was observed in the crystal violet and clonogenic assays. Irinotecan (Figure 9c) also reduced cell survival by inhibiting dehydrogenase activity by −100%, and in this case, the extract showed no effectiveness. This indicates that the extract does not prevent cell damage induced by staurosporine and irinotecan at the level of mitochondrial dehydrogenases. Cisplatin did not cause a reduction in the cell number using this assay and this cell type (Figure 9b).

**Figure 10:**
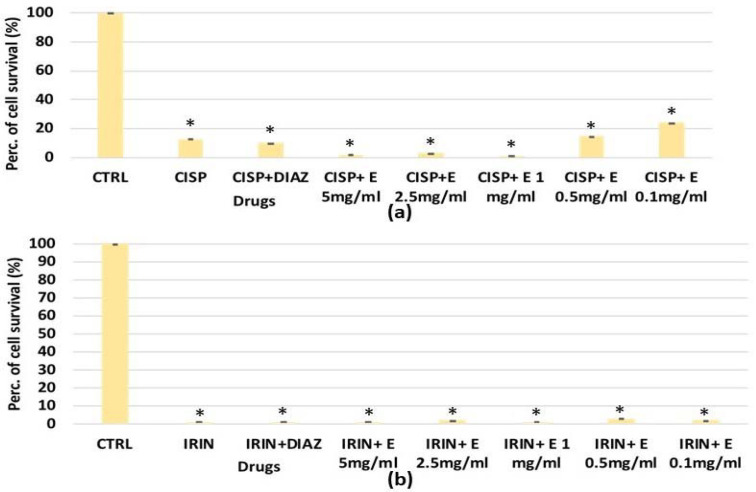
Percentage of cell growth values for SHSY5Y cells co-incubated after 72 h (**a**) with cisplatin (CISP) 1 × 10^−4^ M, (**b**) irinotecan (IRIN) 1 × 10^−4^ M and diazoxide 2.5 × 10^−6^ M (DIAZ) or different concentrations of the extract (E) measured via MTT assay. The reported values represent a mean of at least four replicates. One-way analysis of variance with ANOVA was performed to determine the significance of the data with a value of *p* < 0.05 *.

Similarly, the extract at different concentrations failed to prevent the reduction of cell dehydrogenase activity in the MTT assay performed on SHSY5Y after 72 h of incubation. Cisplatin and irinotecan caused a reduction of −90% and −100%, respectively, compared to the control values (Figure 10a,b).

### 3.4. UV/VIS Spectroscopy Analysis of the Extract’s Composition

**Figure 11:**
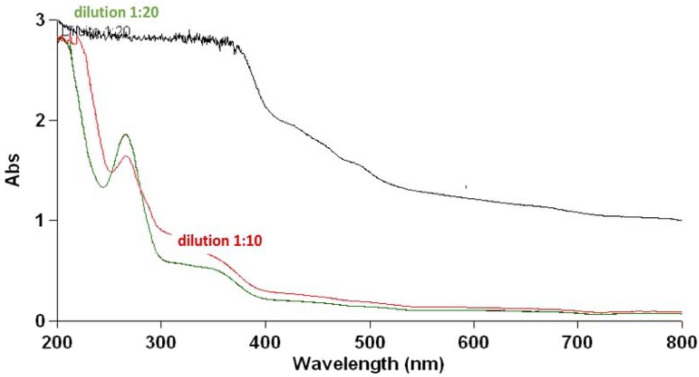
UV/VIS spectrum of a sample of the extract at different dilutions in water. The wavelength expressed in nm is shown on the abscissa axis while the absorbance is shown on the ordinate axis. The sample at a concentration equal to 50 mg/mL, with supersaturation in Abs, is shown in black. In red, is the 1:10 dilution of the starting sample, and in green, is the 1:20 dilution, the black line was the not diluted sample. In both dilutions, the spectrum reported the presence of three peaks at the wavelengths of 350 nm, 260 nm, and 190 nm. The analysis was conducted with Variant 50 Scan. The UV/VIS spectra were used to monitor the stability of the extract during the time.

The analysis was conducted to establish the possible composition of the hydroalcoholic extract to identify the family of compounds responsible for the effects observed. The first sample’s concentration, 50 mg/mL, caused supersaturation in Abs, requiring a 1:10 dilution followed by a 1:20 dilution. The UV/VIS spectroscopy analysis confirmed what was previously reported by Mirali et al., [41], obtaining two peaks at the wavelengths of 350 nm and 260 nm, which correspond to the families of compounds such as flavonoids (quercetin, luteolin, and Kaempferol), proanthocyanidins, which include catechins and procyanidins, and salicylates, such as 4-aminosalicylic acid. We also found a peak at lower wavelength values <200 nm, potentially due to non-aromatic AA (Figure 11). To date, it is still not yet clear which of these molecules (composing the extract) is responsible for these effects.

## 4. Conclusions

In the present work, we evaluated the capability of the novel hydroalcoholic extract from Lens c. in counteracting the reduction of cell proliferation induced by chemotherapeutic drugs and dexamethasone in different cell types. In the cell lines, we found that the extract within a wide range of concentrations (0.01–5 mg/mL) and at different incubation times (6–72 h) prevented the reduction of cell proliferation induced by cisplatin and staurosporine on the renal HEK293 cells measured via crystal violet assay. The extract and diazoxide were also effective in counteracting the staurosporine-induced impairment of the cell colony formations in a clonogenic assay. The extract, however, failed to protect the cells against the cisplatin and irinotecan-induced reduction of cell counts in SHSY5Y neuronal cells.

Diazoxide (250 × 10^−6^ M) was fully effective as a cytoprotective drug on renal HEK293 cells and also effective against dexamethasone-induced osteoblast death. It was also effective against the irinotecan-reduced neuronal cell count, but only after 48 h of incubation time; it was largely ineffective against cisplatin-reduced neuronal SHSY5Y cell counts and bone marrow-induced cell death. This drug is a well-known KATP channel opener that acts on the surface membrane and mitochondrial KATP channels located in the inner mito-membrane. In previous work, diazoxide showed anti-atrophic effects against staurosporine-induced fiber death and neuroprotective effects in humans and animals, and these effects are mediated by either the activation of the surface membrane KATP channels and/or by the inner-mitochondrial KATP channels [11,38]. Ion channels and their associated subunits are indeed emerging as targets that regulate cell cycle and proliferation in different tissues [27,42,43,44].

We believe that the cytoprotective effects of the extract are mediated by the action of cell mitosis and the membrane integrity of the viable cells; indeed, the triarylmethane dye present in the crystal violet assay binds to ribose-type molecules such as DNA in the nuclei and cell membrane target molecules of viable cells rather than at the level of the mitochondrial enzyme.

In native cells, the extract was found to be significantly effective in preventing the reduction of cell proliferation and migration induced by dexamethasone on murine osteoblasts migrating from the isolated bone in the culture. In our experiments, dexamethasone (2 × 10^−6^ M) reduced cell counts by −32% after 24 h of incubation time, and the extract at all concentrations tested was fully effective in restoring the cell count to the control values in the crystal violet assay. The extract prevented the inhibition of mineralization induced by the long-term exposure of the osteoblast culture to dexamethasone. The possible mechanisms of the in vitro actions of the extract and its Phyto complexes can be, indeed, due to interference with the glucocorticoid receptor interaction in osteoclasts, which play a role in proliferation [45] or interference with the dexamethasone induced-decreased synthesis of type I collagen by mature osteoblasts, with the reduced expression of IGF-1 (an autocrine growth factor for osteoblasts), which can be observed in vitro. More recently, several other mechanisms have been proposed, involving miR-22 contributing to dexamethasone-induced osteoblast dysfunction and apoptosis via the miR-22/CAV3 pathway [46] and activating autophagy in osteoblasts [47], or modulating mitochondrial physiology and oxidative stress [48].

Despite the relevant role of the bone marrow mesenchymatous cells in osteoblast genesis, which includes differentiation to adipocytes due to interaction between the nuclear glucocorticoids receptors and the PPARγ2 transcription factor, which, in turn, is needed for adipocyte differentiation [49] or the synthesis of bone-specific proteins, such as the early transcription factor cbfa-1 involved in osteoblastic differentiation in bone marrow [50], the fact that in our experiments the extract failed to prevent the dexamethasone inducing bone marrow depression does not favor the possible involvement of these bone marrow-related mechanisms in the cytoprotective effects of the extract in osteoblasts.

Unfortunately, the extract was not effective in preventing the cytotoxic damage induced by cisplatin, doxorubicin, dexamethasone, and irinotecan on primary murine bone marrow cell cultures at all incubation times and under different experimental conditions. The extract also failed to exert a protective effect against the cytotoxicity induced by doxorubicin and staurosporine on isolated murine skeletal muscle fibers. It should be of note that staurosporine is one of the most effective apoptotic agents inhibiting several tyrosine kinases, including currently known PKC, and doxorubicin is the most powerful cytotoxic drug acting under different mechanisms, including the induction of oxidative stress and alteration of intracellular calcium homeostasis in striated fibers, tumor cells, and bone marrow cells. While cisplatin and irinotecan are chemotherapeutic drugs that selectively act on their targets, dexamethasone is a potent immunosuppressant that also affects osteoblasts. It should be of note that, in our experiments, staurosporine was not effective in reducing cell counts in SHSY5Y neuronal cells despite the well-known role of this agent in inducing apoptosis. SHSY5Y neuronal cells may become resistant to staurosporine cytotoxicity. It may be possible that under our experimental conditions, some genes, like PINK1, can be overactive in protecting the cells against staurosporine-induced apoptosis by impairing the pro-apoptotic cleavage of Beclin1, as was recently reported [51].

This is a novel hydroalcoholic extract, and data on murine cells are not available. The development of the extract under investigation could therefore be placed in the context of research relating to the prevention of hormone-related bone damage by dexamethasone.

Preliminary data suggest that it can be used in the chemoprevention of renal damage from chemotherapeutic alkylating agents, such as cisplatin, and by the broad spectrum of TKI inhibitors, like staurosporine; in this context, further additional investigation is needed.

UV/VIS spectroscopic analysis identified the presence of families of compounds including AA, salicylates, flavonoids, and proanthocyanidins, but in subsequent studies, in which the extraction conditions could be varied, making them selective for one of these categories, they could allow for the identification of the compounds responsible for the observed cytoprotective effects.

The limitation of our observation is that we did not evaluate the cell death pathways involved in the cytoprotective effects of the extract, which is composed of more than 400 molecules acting synergically on cell proliferation and may have the potential to interfere with various cell death pathways. Future works focusing on the effects of the extract on cell mineralization pathways, for instance, are needed to overcome these issues.

## Data Availability

Original data and the product extract tested are available under responsibility of Prof. Domenico Tricarico.

## Supplementary Materials

The following supporting information can be downloaded at: https://www.mdpi.com/article/10.3390/cells11192936/s1, Figure S1: Percentage of cell growth values of HEK293 cells (a) and SHSY5Y (b) co-incubated with different extract concentrations, (E) measured in crystal violet assay after 48 hours of incubation. The reported values represent a mean from at least three replicates. One-way analysis of variance with ANOVA was performed to determine the significance of the data vs. controls (CTRL), with a value of *p* < 0.05 *; Figure S2: Percentage of cell growth values of SHSY5Y (a) after 6 hours of incubation, (b) after 24 h of incubation with different extract concentrations, (E) measured in crystal violet assay. The reported values represent a mean from at least three replicates. One-way analysis of variance with ANOVA was performed to determine the significance of the data vs. controls (CTRL) with a value of *p* < 0.05 *.
