# Supplementary material for: Counteractions of a Novel Hydroalcoholic Extract from Lens Culinaria against the Dexamethasone-Induced Osteoblast Loss of Native Murine Cells
Source: Cells. 2022 Sep 20;11(19):2936. doi: 10.3390/cells11192936 (PMC9563349; doi:10.3390/cells11192936)
Supplement: Supplementary file 1 [file cells-11-02936-s001.zip › cells-1865797-supplementary.pdf]

cells

MDPI

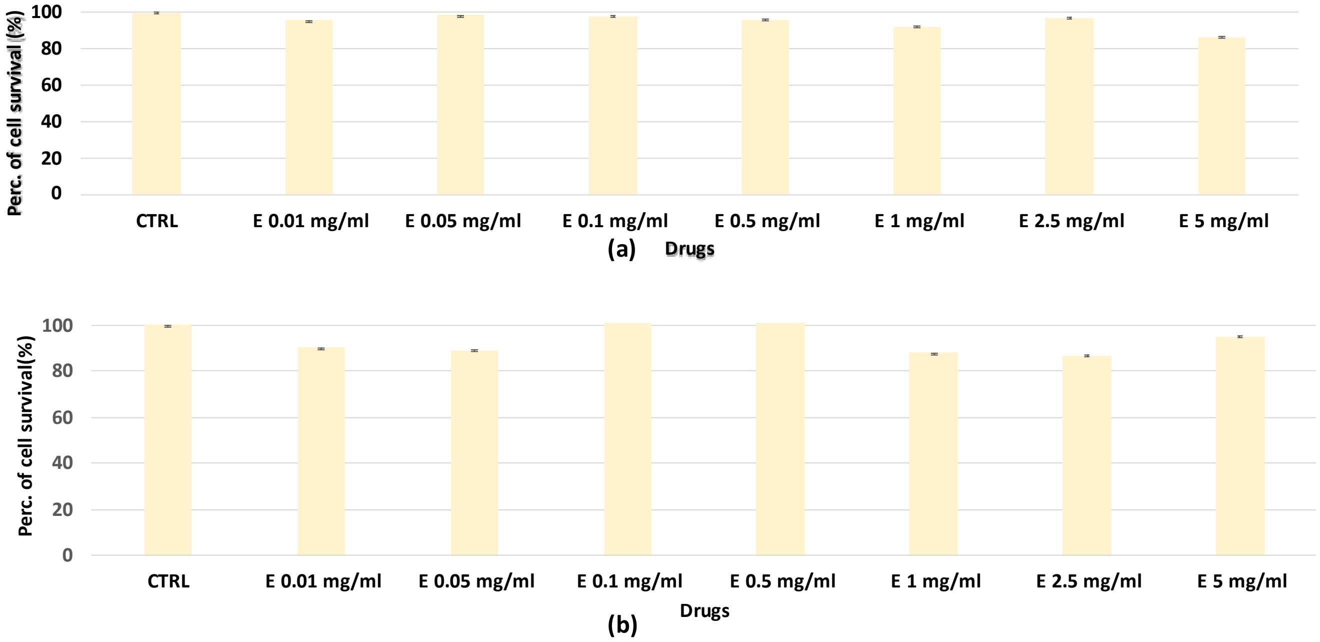

Figure S1. Percentage of cell growth values of HEK293 cells (**a**) and SHSY5Y (**b**) co-incubated with different extract concentrations (E) measured in crystal violet assay after 48 h of incubation. The reported values represent a mean of at least three replicates. One-way analysis of variance with ANOVA was performed to determine the significance of the data vs controls (CTRL) with a value of $p < 0.05$ *.

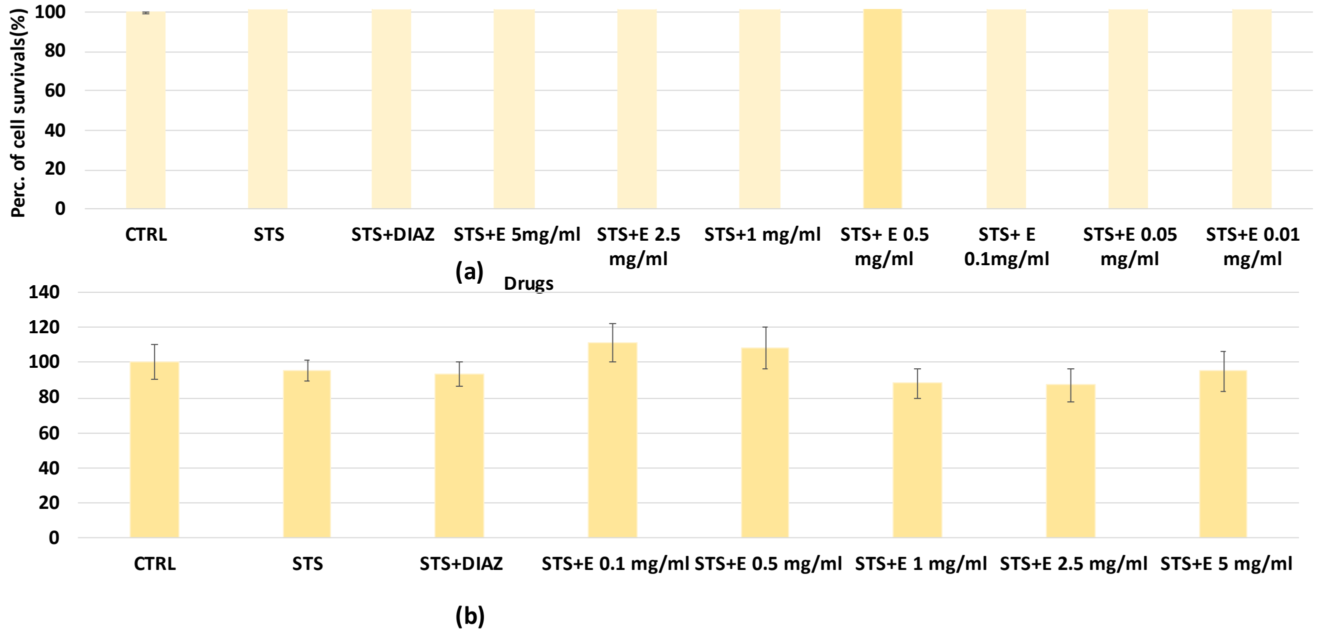

Figure S2. Percentage of cell growth values of SHSY5Y (**a**) after 6 hours of incubation, (**b**) after 24 hours of incubation with different extract concentrations (E) measured in crystal violet assay. The reported values represent a mean of at least three replicates. One-way analysis of variance with ANOVA was performed to determine the significance of the data vs controls (CTRL) with a value of $p < 0.05$ *.
